# Exploring hydrothermal liquefaction (HTL) of digested sewage sludge (DSS) at 5.3 L and 0.025 L bench scale using experimental design

**DOI:** 10.1038/s41598-023-45957-9

**Published:** 2023-11-01

**Authors:** Stian Hersvik Hegdahl, Solmaz Ghoreishi, Camilla Løhre, Tanja Barth

**Affiliations:** Department of Chemistry, University of Bergen, Allégaten 41, 5007 Bergen, Norway

**Keywords:** Biofuels, Environmental chemistry

## Abstract

A common perspective within the prospect of a greener future is utilising our waste materials. One waste material of which the world has abundant resources, and where we will keep having resources, is sewage sludge. This waste material is getting an increased focus, and is commonly utilised by anaerobic digestion processes for methane production. This leaves a bioresidue of digested sewage sludge (DSS). In this study, DSS is submitted to hydrothermal liquefaction (HTL) to produce bio-oil. The studied process includes upscaling as well as considering the effects of temperature, reaction medium of water or ethanol, degree of reactor filling and stirring rate. Promising results are found as high oil yields are obtained also after upscaling. The results reported here show that stirring reduces the need of high temperatures during HTL, providing energy savings that are promising for further upscaling. In addition, a total of 18 compounds are identified and semi-quantified, showing an abundance of fatty acids and fatty acid derivatives within the oil, encouraging further studies towards separation of said fatty acids for use as biodiesel.

## Introduction

Hydrothermal liquefaction (HTL) is a process in which biomass is converted to a bio-oil at high temperatures (250–400 °C) and at high pressure (at or above the steam saturation pressure at the chosen temperature)^1^. Feedstocks with high water content is the primary choice for HTL since drying is not required, as for other thermochemical processes, such as pyrolysis or gasification which require dry materials as input. Feedstocks that have been used include lignocellulosic biomass, algae, fish sludge, sewage sludge or food wastes^2–6^. The dominating reaction pathway is hydrodeoxygenation, which removes oxygen and adds hydrogen to the reaction products during depolymerisation of biopolymers. Inclusion of formic acid during HTL has shown good effects as a hydrogen donor which significantly increases the yields of bio-oil^7^.

This study addresses HTL of the residue after anaerobic digestion of sewage sludge (digested sewage sludge, DSS). This is a underutilised, organic-rich waste, which contains considerable amounts of energy in the form of organic residues and microbial biomass even after generation of methane by anaerobic fermentation. Presently, the major use of DSS is as soil improver and fertiliser. This use is limited by a number of factors^8^, including the risk of pollution by organic and inorganic constituents in the DSS and the need for transport to suitably large areas of arable land^9^, which increases the costs. The growing number of biogas plants also gives increasing volumes of DSS that have to be handled. As valorisation of biomass residues and wastes is a major focus for developing a circular economy^10–12^, in the perspective of the European waste hierarchy, the optimal use of such material is energy recovery, since it cannot be reused^13^.

There are a number of studies using sewage sludge as feedstock for HTL conversion to biooil, but few specifically address DSS. There is a significant chemical difference in the DSS compared to untreated sewage sludge due to the increased content of microbial biomass, e.g. a DSS sample has been measured to contain 14.92% N (w%) compared to 3.7% in the untreated sludge^14^, reflecting an increased content of proteins from the microbial biomass. Published results indicate that DSS is a very suitable feedstock for HTL: Aragon-Briceno et al.^14^ and Aragon-Briceno et al.^15^ published a study focussing on DSS and the integration of hydrothermal treatment with a wastewater treatment plant and the increase in use of product value after this process, with hydrochar yields of up to 56%. An alternative approach is described by Stutzenstein et al.^16^ using hydrothermal carbonisation on the DSS in a 1.2 L HTC reactor getting dry mass yields of up to 49 wt% with feedstocks including general waste products and digestate (DSS)^17^. Saba et al.^18^ reported results on mixing DSS and lignocellulosic feedstock in a co-HTL processing. On a more general HTL-focus, Leng et al.^19^ reports retrieval or mitigation on the bio-oils produced based on feedstock that generally provide higher nitrogen contents, such as DSS, and Xu et al.^20^ shows fractionation of phosphorus from sewage sludge and sludge ash. Both Madsen et al.^21^ and Sudibyo et al.^22^ have provided insight as to the origin of common chemical structures found in the HTL liquids based on the normal compound classes present within biomass, such as lignin, cellulose, lipids and proteins.

Our previous screening study of parameters for HTL of DSS performed in small laboratory scale using a temperature, time and KOH as catalyst as experimental variables, provided promising results of up to 58 wt% oil yields, (dry, ash free basis) (*daf*), with oils containing useful compounds such as fatty acids, aliphatic alcohols, glycols and phenols^23^. A logical next step towards HTL industrialisation is upscaling to larger scale to provide bio-oil volumes for realistic testing, as has been done before by Ghoreishi et al.^24^ using lignin as feedstock.

In HTL, water is commonly used as reaction medium, yet, in previous work on other feedstocks, ethanol has been used as an alternative with considerable success^7,25,26^. Though water has obvious advantages in being a cheap and abundant reaction medium, ethanol has provided higher bio-oil yields for lignocellulosic^27^ and lignin based feedstocks^28^ and sewage sludge^29^, and is therefore included as an alternative reaction medium in this work. The use of ethanol in the reaction medium makes the mass balance and energy recovery calculations more complex^30^, since previous work on lignin has shown some incorporation of carbon from formic acid in the aromatic molecules in the produced bio-oil^31^, and also shown that alkyl groups from the alcohols are found as substituents on aromatic rings in the bio-oil product^32^. The contribution from such incorporations must thus be considered in the final selection of optimal conditions.

In this paper, the effect of upscaling from 0.025 to 5.3 L reactor volumes is systematically screened with regards to biooil yields and energy recovery in the organic phase, and the most abundant compounds in the bio-oils are identified. The study has a local perspective, as the feedstock has been supplied by the municipal unit Bergen Water from their biogas facility in Bergen, Norway. An experimental screening design and multivariate analysis for interpretation have been used to evaluate the effects of the experimental conditions on these outcomes. Multivariate analysis is also used to compare results obtained in a 5.3 L reactor with a corresponding experimental series performed in a 25 mL reactor. Combining the two experimental series, the total set of variables are reactor size (25 mL and 5.3 L), temperature (280 and 380 $^\circ{\rm C}$), reaction medium (water or ethanol), volume of the reaction medium (3 and 6 mL for small scale, 450 and 900 mL for large scale) and stirring rate (200 and 1000 rpm, only applicable for large scale). The results of this study show promising oil yields and energy recoveries, as well as identification of the most abundant compounds within the oils. We also perform a partial least squares (PLS) modelling that provides equations that estimate the effect of the experimental factors on the product yields within the parameter values that are tested.

## Materials and methods

The dewatered DSS feedstock used in this study was provided by Bergen Water and their biogas facility in Norway. The DSS was used as received with no drying or homogenisation, hence there is a risk of a certain degree of inhomogeneity in the batches used for each experiment.

Ethyl acetate (EtOAc, > 99.5%), ethanol (absolute, 99.96%), formic acid (> 98%), sodium sulphate ($\ge$ 99.0%, anhydrous), dodecane (analytical standard), benzoic acid ($\ge$ 99.5%), pyridine ($\ge$ 99.5%), *N*,*O*-bis(trimethylsilyl)trifluoroacetamide (BSTFA) w/1% trimethylchlorosilane (TMCS)) and pentane ($\ge$ 99%) were purchased from Merck (Saint-Louis, MO, USA) and used without further purification.

The oil yields are calculated by mass of oil produced relative to input mass of dry, organic feedstock. The energy recovery in the bio-oil is calculated relative to the organic matter input in the DSS. As formic acid does not contribute significantly to the oil yield^31^, this input is not included in the yield calculations. When ethanol is included in the reaction medium, it can react with specific hydroxyl substituents^32^ or in esterification of carboxylic acids. However, such contributions were not included in the yield calculations due to challenges in quantification, and this gives an unknown uncertainty in the recovery values for the different systems.

### Large scale conversion

Non-dried DSS (600 g), formic acid (150 mL) and water or ethanol (450–900 mL) were added to a 5.3 L high pressure reactor from ESTANIT GmbH, before closing and heating to 280–380 °C at a stirring rate of 200–1000 rpm. Heating was performed with a heating rate of 4.4–4.8 $\mathrm{^\circ{\rm C} }$ min (after loading), and 4 h of residence time (measured after reaching the desired temperature). Cooling of the reactor was done by cold water in a cooling coil inside the reactor, maintaining the stirring speed, until the following day. The gaseous phase was vented, and to ensure sufficient filtration, the contents were filtered threefold using Whatman 589/3 filter papers and EtOAc as reaction medium. The aqueous and organic phases were separated by decanting, and the aqueous phase was extracted using EtOAc until the organic phase from the extraction was clear. The total organic phase was dried over sodium sulphate until the next day and evaporated on a rotary evaporator (40 °C, 200 mbar for experiments using water as reaction medium, 40 $^\circ{\rm C}$, 160 mbar for experiments using ethanol as reaction medium).

### Small scale conversion

Non-dried DSS (4 g), formic acid (1 mL) and water or ethanol (3–6 mL, giving a DSS concentration of 1.33–0.67 g/mL, respectively) were added to a 25 mL 4740-series Parr reactor, which was closed and heated to 280–380 °C for 4 h in a preheated Carbolite Laboratory High Temperature oven. After cooldown to ambient temperature, the gaseous phase was vented, and the contents were filtered by filter paper (Ahlstrom-Munksjö) followed by glass fibre filters (Whatman, GF/A) to recover the solid phase. EtOAc was used as reaction medium during the work-up. The aqueous and organic phases were separated by decanting before the organic phase was dried over sodium sulphate for 1 h and evaporated on a rotary evaporator (40 °C, 200 mbar for experiments using water as reaction medium, 40 $^\circ{\rm C}$, 160 mbar for experiments using ethanol as reaction medium).

### Experimental design

The experimental design was set up as two blocks, one for the large scale experiments and one for the small scale experiments. The large scale experiments were set up as a 2^4–1^ fractioned factorial design^33^, with four variables. These were temperature (280–380 $\mathrm{^\circ{\rm C} }$), water or ethanol as the reaction medium (a 50/50 mixture was used for the centre point), degree of filling (450–900 mL, giving a DSS concentration of 1.33–0.67 g/mL, respectively) and stirring rate (200–1000 rpm). Two experiments were performed using centre point values, and two experiments were repeated for the experiment providing the highest oil yields.

In small scale, the experiments were set up as a 2^3^ full factorial design^33^. Stirring is not available in the small scale reactor, and so this variable was not included. The three variables were therefore temperature (280–380 $^\circ{\rm C}$), water or ethanol as the reaction medium (a 50/50 mixture was used for the centre point) and degree of filling (3–6 mL). Three experiments were performed using centre point values.

The full, merged design is given in Table 1. All experiments were run in a randomised order individually for each block.

**Table 1:** Experimental variables showing the conditions used for each experiment in large- and small scale.

Reactor size	T (^o^C)	Reaction medium	Filling^a^ (mL)	Stirring (rpm)	Conditions^b^	Experiment^c^
Large	280	H_2_O	450	200	+ − − − −	L.280.H2O.450.200
Large	380	H_2_O	450	1000	+ + − − +	L.380.H2O.450.1000
Large	280	EtOH	450	1000	+ − + − +	L.280.EtOH.450.1000^d^
Large	380	EtOH	450	200	+ + + − −	L.380.EtOH.450.200
Large	280	H_2_O	900	1000	+ − − + +	L.280.H2O.900.1000
Large	380	H_2_O	900	200	+ + − + −	L.380.H2O.900.200
Large	280	EtOH	900	200	+ − + + −	L.280.EtOH.900.200
Large	380	EtOH	900	1000	+ + + + +	L.380.EtOH.900.1000
Large	330	H2O/EtOH	675	600	+ 0 0 0 0	L.330.H2O/EtOH.675.600^d^
Small	280	H2O	3	–	− − − −	S.280.H2O.3.NS
Small	380	H_2_O	3	–	− + − −	S.380.H2O.3.NS
Small	280	EtOH	3	–	− − + −	S.280.EtOH.3.NS
Small	380	EtOH	3	–	− + + −	S.380.EtOH.3.NS
Small	280	H_2_O	6	–	− − − +	S.280.H2O.6.NS
Small	380	H_2_O	6	–	− + − +	S.380.H2O.6.NS
Small	280	EtOH	6	–	− − + +	S.280.EtOH.6.NS
Small	380	EtOH	6	–	− + + +	S.380.EtOH.6.NS
Small	330	H_2_O/EtOH	4.5	–	− 0 0 0	S.330.H2O/EtOH.4.5.NS^e^

^a^Filling indicates the amount of the reaction medium only.

^b^The + , 0 or − notation indicate whether the factors are in their high ( +), centre (0) or low (−) values.

^c^The experiments are coded by reactor size (S = small, L = large), temperature, reaction medium, degree of filling and stirring rate (NS = nonstirred).

^d^This experiment was performed twice.

^e^This experiment was performed threefold.

#### Multivariate data analysis

A Partial Least Squares (PLS) regression analysis was performed using the program Sirius 11.0. Cross factor interactions were included as experimental variables in the multivariate data analysis. The chemometric models give an equation that estimates the selected output (e.g. oil yield) based on the variables used^33^. Initially, all factors and cross factors are included in the regression models, and then the impact of each factor on the regression equation is individually evaluated. The interactions between different factors may be as important as the factors themselves, hence cross factors are considered in the regression analyses. Factors that have an insignificant impact on the model for each output are removed from the equation. This is monitored by studying the R^2^-values after removal of the factors.

However, the large scale experimental series is done as a 2^4–1^ fractioned factorial design, so each cross factor will overlap with one other cross factor, hence only being able to provide a sum of the two overlapping factors. Evaluating the relative importance of the factors in the large scale design is done by comparison with their effects in the full design for the small reactor, and also by analysing the merged dataset from the two experimental series^33^.

The statistical significance of the correlations is evaluated from the correlation coefficient and the root mean square error of cross-validation (RMSECV) of the PLS models^33–35^.

### Silylation

All oils were silylated prior to GC–MS analysis. This was done by dissolving the oil (0.01 g) in EtOAc (3 mL), containing benzoic acid and dodecane as internal standards (0.017 mg/mL each). The solution (1 mL) was transferred to a new vial, adding pyridine (0.150 mL) and *N*,*O*-bis(trimethylsilyl)trifluoroacetamide (BSTFA) (0.150 mL) to the mixture. The vials were capped and heated (70 °C, 30 min) before transfer to a new vial (0.700 mL), mixing with pentane (0.700 mL). The final solution contains approx. 1.5 mg/mL of the silylated oil and 6.5 μg/mL of each internal standard. The samples were cooled at 5 °C overnight and filtered through a 0.45 μm Puradisc NYL filter before GC–MS analysis.

### GC–MS

All oils were analysed by GC–MS after silylation on an Agilent Tehnologies 7890 A GC with auto-sampler, using an Agilent 5977 A MSD. 1 µL injection volume and splitless mode was used, with an injection temperature of 280 °C and the auxiliary heater at 300 °C. The column was an Agilent Technologies 30 m HP-5ms with a thickness of 0.25 μm and ID of 250 μm. A carrier gas velocity of 1 mL/min was used, and the temperature program was as such: 50 °C * 2 min; 10 °C/min to 200 °C; 20 °C/min to 300 °C * 5 min.

The MS detector has an ion-source temperature set to 230 °C, spanning from 25–400 u with a scan speed of 1.56 *mz*/s and a positive ionisation of 70 eV. The solvent delay was at 5.50 min. Identification was performed using Enhanced Data Analysis, MSD ChemStation, which is coupled to the NIST 2.0 library.

### Elemental analysis

The oils were analysed by two parallels in CHNS-mode, using a Vario El III, with He as the carrier gas. Oxygen is calculated by difference. The instrument is not calibrated to measure sulphur.

The results from elemental analysis are used to calculate the higher heating value (HHV) and energy recovery. The HHV is calculated by the formula presented by Channiwala et al. (2002)^36^. The energy recovery is calculated by $HHV\times m$ for the oil divided by $HHV\times m$ for the feedstock, where *m* represents mass of the oil from HTL and mass of the feedstock used in the experiment, respectively.

## Results and discussion

### Large scale experiments

#### Feedstock

Determination of the bulk composition of the feedstock in the large scale experiments was performed by the biogas facility (Bergen Water) and is given in Table 2, together with the elemental composition of the organic fraction. The ash content is quite high as nearly half of the dry weight is inorganics.

**Table 2:** Feedstock information.

Feedstock	DSS for large scale
Date received	14.01.2021
$Dry solid content {\left(wt\%\right)}^{a}$	26.4
$Ash {\left(wt\%\right)}^{a}$	11.4
$$Dry, ash free organic content \left(wt\%\right)$$	15.0
${C}_{dry} \left(wt\%\right)$	31.4 $\pm$ 0.3
${H}_{dry} \left(wt\%\right)$	4.7 $\pm$ 0.4
${N}_{dry} \left(wt\%\right)$	4.1 $\pm$ 0.7
${O}_{dry} {\left(wt\%\right)}^{b)}$	16.4 $\pm$ 0.6
${H/C}_{molar ratio}$	1.8 $\pm$ 0.2
${O/C}_{molar ratio}$	0.4 $\pm$ 0.0
${N/C}_{molar ratio}$	0.1 $\pm$ 0.0
$HHV {\left(\mathrm{MJ}/\mathrm{kg}\right)}^{c}$	13.8 $\pm$ 0.5

^a^Values received from Bergen Water (personal communication, 2021).

^b^Oxygen content was found by difference.

^c^HHV is calculated from dried feedstock, based on the formula presented by Channiwala et al.^36^.

#### Oil yields

**Figure 1:**
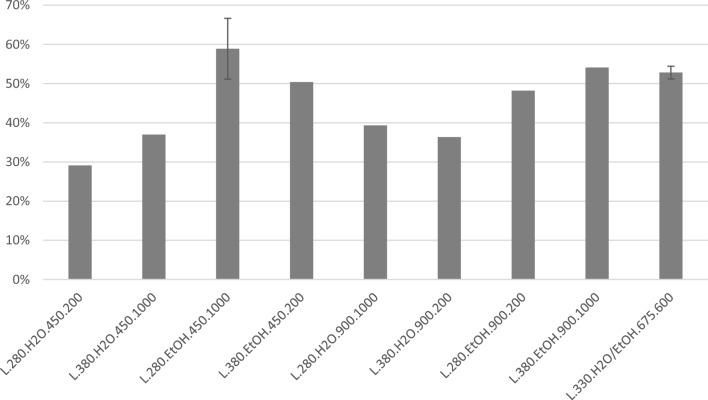
Yields of oil from large scale experiments given as % mass relative to organic biomass input (dry, ash free basis). For experiments with replicates, the average is shown with the high and low values given as uncertainties. The experiments are coded by reactor size, temperature, reaction medium, degree of filling of reaction medium, and stirring rate.

The bio-oil yields are shown in Fig. 1 as organic compound mass % relative to organic biomass input (on a dry, ash free mass basis).

As shown, the highest yield obtained in large scale (67%) was obtained from experiment L.280.EtOH.450.1000 (+ − + − +). A replicate of this experiment provided an oil yield of 51%. The parallel centre-point experiments in large scale, L.330.H2O/EtOH.675.600 (+ 0 0 0 0) provided oil yields of 51 and 54%. The variation between the parallel experiments can be caused by non-homogenous feedstock, since the DSS has been used as received without further homogenisation. Even with such variations, these oil yields are higher than previously reported in literature (9.4% from DSS^5^; 34.4 wt% *daf* from DSS^37^; 28.0 wt% from sewage sludge^38^; 58 wt% *daf* from DSS^23^).

#### Elemental composition and higher heating value

**Figure 2:**
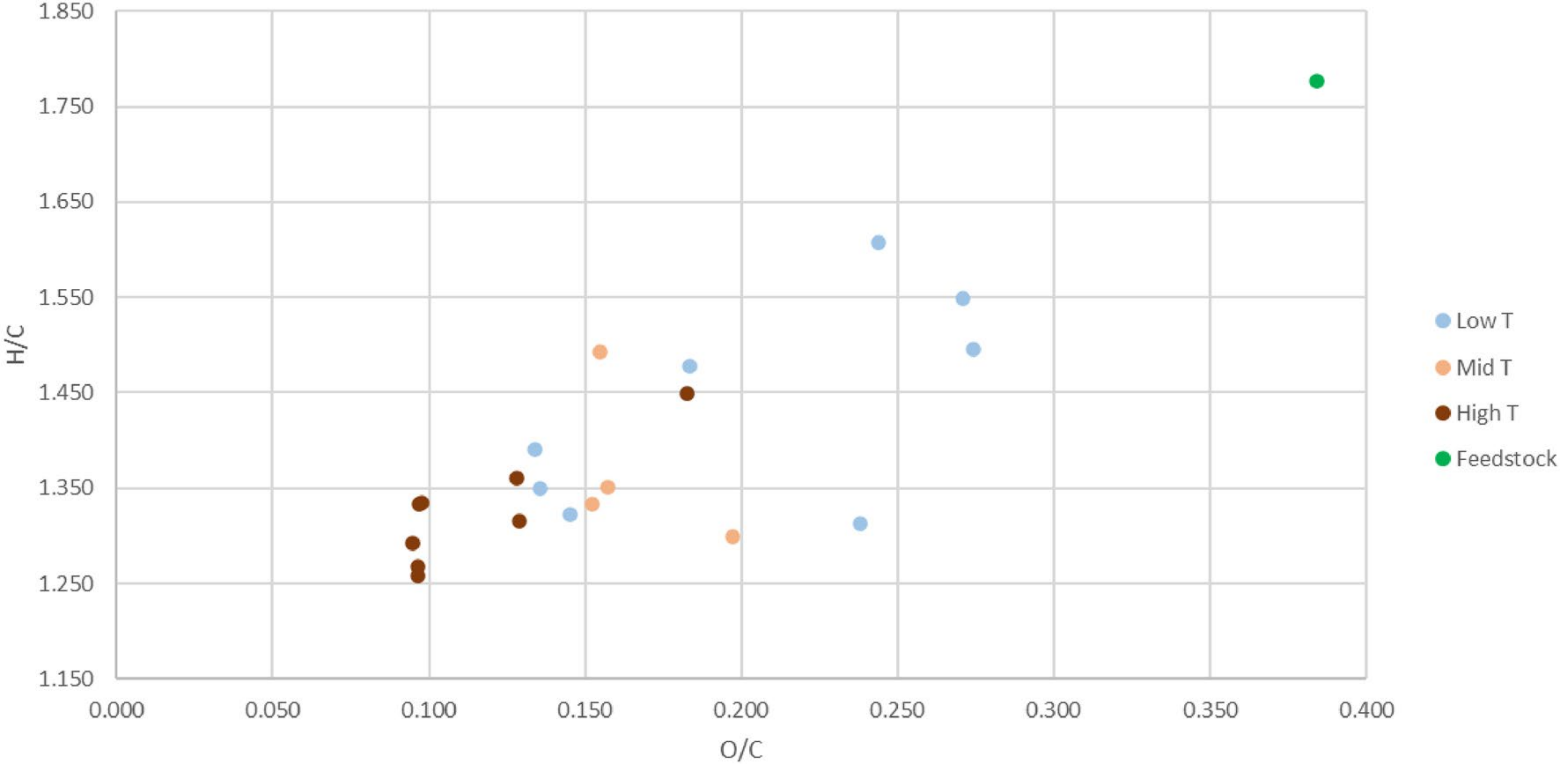
Van Krevelen diagram of oils, color-coded by temperature.

Figure 2 shows a Van Krevelen diagram in which the hydrogen to carbon molar ratio is plotted against the oxygen to carbon molar ratio. The bio-oils show a trend as a function of the reaction temperature, as higher reaction temperatures provide both lower O/C-ratios and H/C-ratios. All in all, both O/C and H/C are reduced in large scale compared to the feedstock (in green). This suggests that elimination of water from the organic components could be an important reaction in the thermochemical conversion even under hydrothermal conditions.

**Figure 3:**
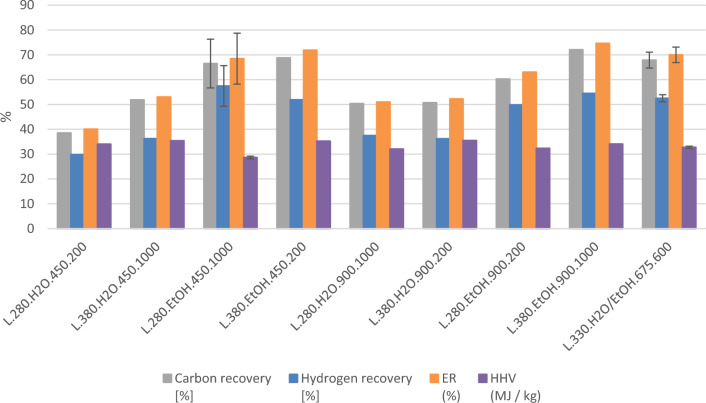
Elemental composition of the bio-oils, together with the energy recovery and higher heating values^36^ of the bio-oils. The experiments are coded by reactor size, temperature, reaction medium, degree of filling of said reaction medium, and stirring rate.

Figure 3 shows the HHV, energy recovery and carbon and hydrogen recoveries. All the graphs show error bars for two of the experiments, which are the experiments performed in parallel. Average values are given in the graphs, with the high and low values given as margins of error.

As would be expected, the graphs for hydrogen recovery, carbon recovery and energy recovery show similar trends, see Fig. 3. All of them show a significant difference between the experiments as a function of reaction medium. The experiments containing ethanol as reaction medium give significantly higher recoveries than the ones using water. The centre point experiment with 50% ethanol, 50% water as reaction medium fit into the trend for energy recovery seen in the experiments containing ethanol. The observation that a mixture of an alcohol and water as reaction medium gives the highest energy recovery is in line with published results for SS^27,29,30^. Further investigations would be needed to determine the specific reactions that cause the higher energy content of bio-oils produced with ethanol as part of the reaction medium.

### Small scale experiments

#### Feedstock

The feedstock for the small scale experiments were received from the biogas facility in Bergen (Bergen Water), who also provided the results from gravimetric analysis given in Table 3. No results from elemental analysis are given for this feedstock, due to lack of reliable measurements. The organic content is comparable to the feedstock for the experiments in the large reactor, so the feedstocks are considered suitable for comparison.

**Table 3:** Feedstock information.

Feedstock	DSS for small scale
Date received	27.04.2020
$Dry solid content {\left(wt\%\right)}^{a}$	27.6
$Ash {\left(wt\%\right)}^{a}$	11.3
$$Dry, ash free content \left(wt\%\right)$$	16.3

^a^Values received from Bergen Water (personal communication, 2020).

#### Oil yields

**Figure 4:**
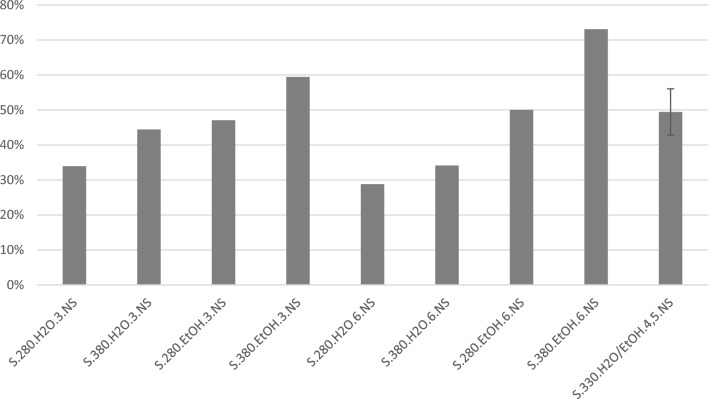
Yields of oil from small scale on dry, ash free basis, given as % mass relative to organic input. For experiments with replicates, the average is shown with the high and low values given as uncertainties. The experiments are coded by reactor size, temperature, reaction medium, degree of filling of said reaction medium, and stirring rate (NS, non-stirred).

Figure 4 shows the oil yields from small scale experiments. In small scale, the maximum oil yield (73%) was obtained from experiments with variables set to their highest value and ethanol as reaction medium. When comparing with the large scale experiments given in Fig. 1 the same trends can be seen; a significant change in the oil yield is seen when using ethanol as reaction medium instead of water, with all experiments with ethanol in the reaction medium providing higher oil yields than the comparable experiments using water. There are some minor differences relative to the large scale experiments in that the highest oil yield at small scale was obtained at high temperature and degree of filling, while these variables were at their low value for the highest oil yield in the large scale experiments. Despite these minor differences, the same experiments provided overall high oil yields for the two scales, showing a general consistency within the experiments even when working at different scales.

### Regression models for both scales and combined data

#### Partial least squares (PLS)

Results from multivariate screening designs are not suited for direct interpretation, and need a corresponding multivariate data analysis approach to give systematic and quantitative relationships for further interpretation. For this purpose, a PLS regression analysis was performed on the results from both scales to establish equations that describe the oil yields as a function of the experimental variable values. These equations are given in Table 4. The fit of the models to the data is given by the correlation coefficient R^2^, which in all cases is higher than 0.89, and of the RMSECV, which is no higher than 7.3. These data show a strong systematic variation in the designs^33,34^. In the final equations, parameters that did not contribute to increasing the value of the regression have been removed from the final equation.

**Table 4:** Regression equations, showing the correlations between the variables and the yields.

Factor	Regression equation^a^	R^2^	RMSECV
Y_oil_ (small scale)	$46.98 + 6.413T + 11.04M + 4.012MF$	0.927	7.34
Y_oil_ (large scale)	$45.13 + 8.725M + 3.150S - 2.075\left( {TS + MF} \right)$	0.904	5.69
Y_oil_ (comb. scale)	$46.06 + 3.351T + 9.867M + 4.281S - 3.052ReT - 4.312TS + 2.642MF$	0.894	6.05

^a^Re, Reactor size; T, Temperature; M, reaction Medium; F, degree of Filling; S, Stirring rate; R^2^, coefficient of determination; RMSECV, Root Mean Square Error of Cross-Validation.

**Figure 5:**
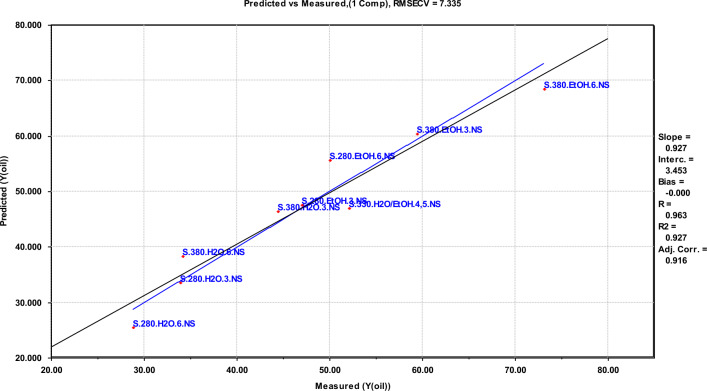
Predicted versus measured oil yields in small scale.

The first equation in Table 4 shows a positive correlation between the oil yield in small scale and both temperature (T), reaction medium (M) and the cross factor of reaction medium (M) $\times$ degree of filling (F). The highest oil yield is thus obtained at a high temperature, with ethanol as reaction medium. Since stirring is not an option at small scale, this factor cannot be evaluated. A graph showing the predicted vs. measured oil yields is given in Fig. 5.

**Figure 6:**
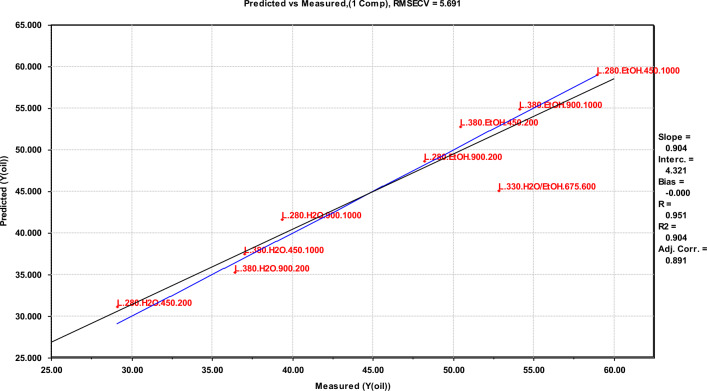
Predicted versus measured oil yields in large scale.

In the large reactor, a direct correlation between temperature and oil yield is no longer seen (second formula of Table 4), however using ethanol as the reaction medium still increases the oil yield, which is also the case for increasing the stirring rate. Lastly, there is a combined effect of the cross factors temperature (T) $\times$ stirring (S), and reaction medium (M) $\times$ filling (F). The combined effect of these cross factors (TS + MF) has a negative correlation to the oil yield. Due to the positive effect from the cross factor in the small scale reactor an assumption can be made that the cross factor between temperature and stirring has an even larger negative effect. Predicted vs. measured oil yields for large scale are shown in Fig. 6.

**Figure 7:**
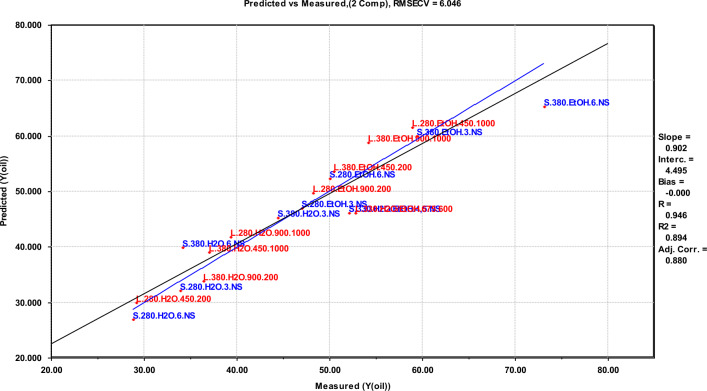
Predicted versus measured oil yields for both scales combined. Blue colors represent small scale experiments while red colors represent large scale experiments.

By merging the two experimental series into one large series, a larger dataset is acquired, allowing each cross term to be studied individually (third formula of Table 4). The estimated oil yield based on the combined scales still shows positive correlations from both temperature, reaction medium and stirring rate to the oil yield, as well as for the cross term between reaction medium (M) and filling grade (F). Interestingly, also two negatively correlating cross terms are shown, which is the cross terms reactor size (Re) $\times$ temperature (T) and temperature (T) $\times$ stirring rate (S). This implies that a lower temperature setting will increase the oil yield if a larger reactor and a higher stirring rate is used, allowing the user to reduce the energy input while increasing the oil yield. Predicted vs. measured oil yields for the merged series are given in Fig. 7.

The cross term of reactor size (Re) $\times$ temperature (T) might be explained by the lack of stirring in the small scale reactor. If stirring had been possible in small scale, this might have eliminated the $\mathrm{Re}\times \mathrm{T}$ cross term, increasing the effect of the cross-factor $\mathrm{T}\times \mathrm{S}$. This would however require further experiments to validate.

For the oil yield in large scale, the centre points deviate from the predictions, as seen in Fig. 6. This also goes for the oil yield in combined scale (Fig. 7). This implies a non-linear regression within the data range covered. In this data set, there are two potential factors explaining this: (1) An assumption is that the reaction medium is key to the non-linearity since a 1:1 ratio is not a centre point when two substances are mixed. There could be many ways to select a centre point between 100% water and 100% ethanol, e.g. volume, mass, polarity, etc.; (2) Cracking of bio-oil has a significant effect on oil yields at high temperatures, giving a maximum oil yield and then a decrease at higher temperature levels^24,39^. Both squared and logarithmic terms of all main variables were incorporated in the PLS-dataset, however none of these proved significant or included the centre values. Further investigation would require an additional experimental design, in which the medium is gradually changed from water with 0% ethanol to ethanol with 0% water.

In general, the equations suggest that the highest possible oil yield are obtained at a low temperature, combined with a high stirring rate, allowing both the main factor of the stirring rate, as well as the cross term of TS to contribute to a higher oil yield. To maximise oil yields, a high volume of ethanol should be used, the main factor for reaction medium, as well as the cross term of MF contributing to a higher oil yield.

The fractional factorial experimental design is suitable for use in screening the importance of the selected experimental parameters. More experiments in an extended design would be needed to determine the definitive optimal process conditions for upscaling. The deviations between parallel experiments are one cases quite large—16% at the conditions giving a maximum oil yield. The difference is most probably explained by variability in the DSS, as it is a complex mixture of original constituents in the sewage sludge and bacterial biomass from the anaerobic digestion. However, the regression models shown in Figs. 5, 6 and 7 have a god fit and a high correlation coefficient for the predicted versus measured line, and establishing separate models excluding one parallel at a time does not significantly change the coefficients in the equations or the fit of the model. This supports the evaluation that the observed trends are statistically significant and can be used to evaluate the importance of the reaction parameters that have been investigated in the context of a screening study.

Taking economic factors into account, the use of ethanol may not be the preferred option, and it is thus of interest to evaluate the results when using only water as the reaction medium. The highest oil yield is then 44 wt% for small scale and 39 wt% for large scale. Both are significantly lower than the 73 wt% in small scale and 69 wt% in large scale obtained using an ethanol-system. As the lowest yields with ethanol are higher than the highest yields with water, a concept including ethanol as reaction medium during HTL and recycling of unreacted ethanol after workup should still be considered. However, a more extensive techno-economic evaluation would be needed for a final conclusion on the use of ethanol or not.

#### Molecular composition of the oils

**Figure 8:**
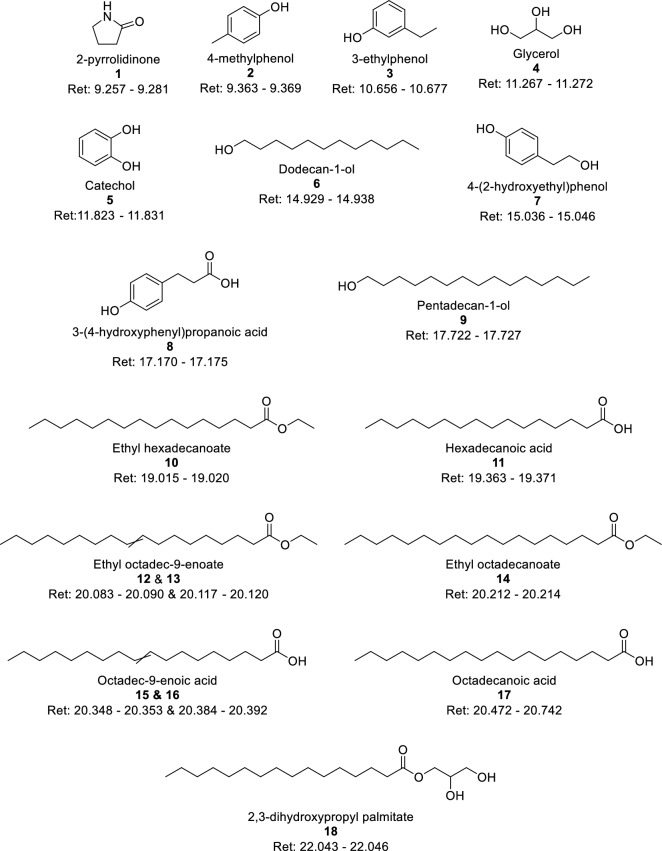
Structure overview, providing compound identification, with names and retention times in minutes, for Table 5.

**Figure 9:**
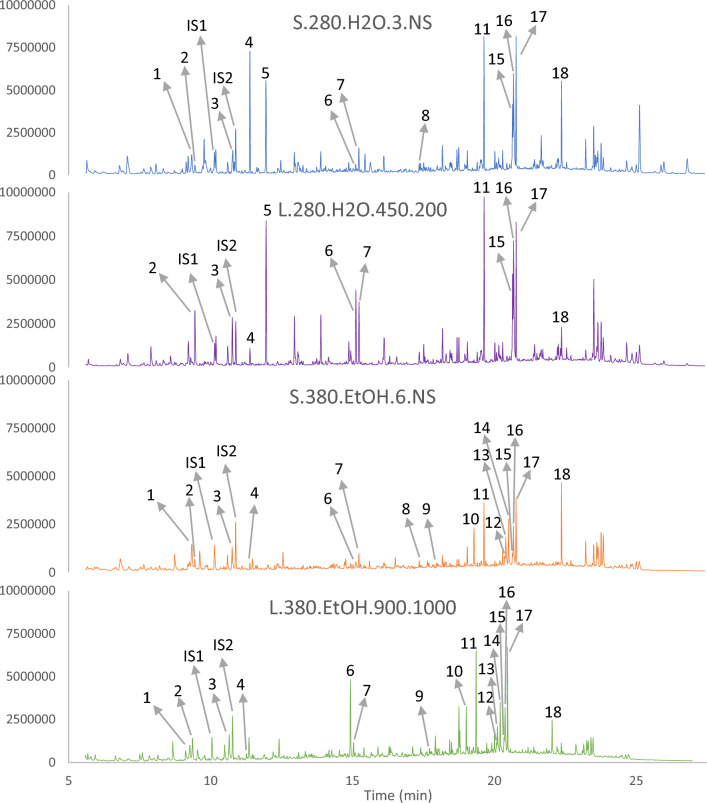
Gas chromatograms of selected bio-oils. The illustrated oils include S.280.H2O.3.NS (− − − −), L.280.H2O.450.200 (+ − − − −), S.380.EtOH.6.NS (− +  + +) and L.380.EtOH.900.1000 (+ +  +  + +). Compounds are indicated as defined in Fig. 8. IS1 = internal standard no. 1, which is dodecane. IS2 = internal standard no. 2, which is benzoic acid.

Figure 8 and Table 5 provide an overview of the most abundant compounds within the oils while Fig. 9 shows chromatograms of selected oils. The values are presented as peak areas relative to the internal standard, and thus can be compared only as relative variations between samples and not as absolute concentrations since the response factors for each compound is not known. As is seen clearly from Fig. 9, there are significant differences in specific compound abundance between the oils. Table 5 shows the top 5 largest compound peaks identified within the oils with a peak size above $3\times {10}^{7}$, converting to roughly 1–10 µg/mL within the GC solution, depending on the response factor. Note that some of the oils have only 4 compounds marked as the top 5. In these cases, different unidentified sterols are the fifth peak. Some of the compounds are present in similar concentrations in all samples (3-ethylphenol, **3**; catechol, **5**; ethyl octadec-9-enoate, **12**; octadec-9-enoic acid, **15**), while there is significant variation in the amounts of hexadecenoic acid (**11**), octadec-9-enoic acid (**16**) and octadecanoic acid (**17**), representing the largest peaks in the majority of the oils.

**Table 5:**
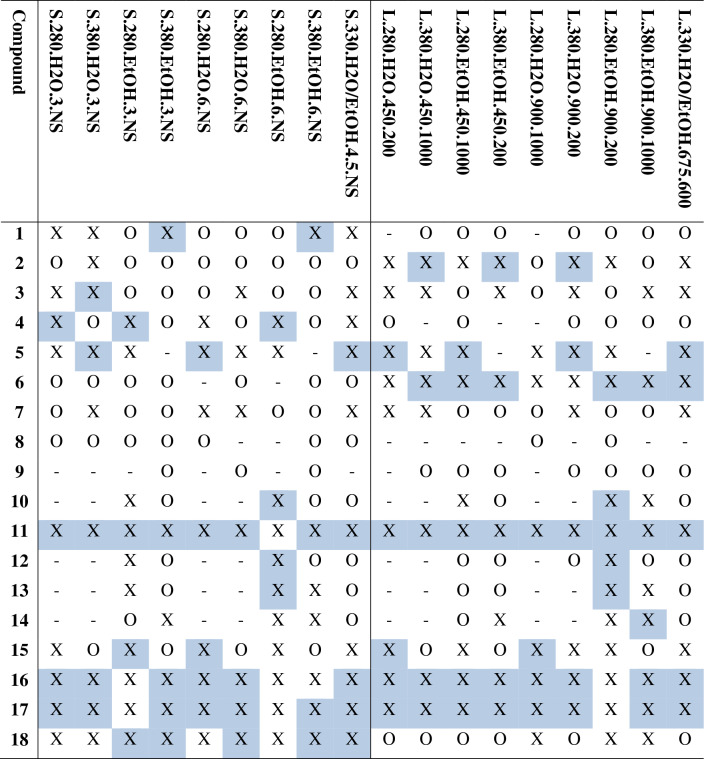
Compound classification of peaks within the bio-oils, selected based on peak abundance as well as compounds differing between scales.

Structures are defined in Fig. 8.

O = Peak found in the chromatogram; X = Peak found in the chromatogram, with an area larger than $3\times {10}^{7}$; Blue background indicates the compound being within the top 5 largest peaks of the bio-oil.

When ethanol is used as reaction medium, the fatty acids are partly esterified. In these cases, both the fatty acids and their respective fatty acid methyl esters (ethyl hexadecanoate, **10**; ethyl octadec-9-enoate, **13**; ethyl octadecenoate, **14**) show as high peaks, emphasizing the abundance of these compounds in general and their ability to form ethyl esters with the ethanol that is present. Such incorporation of the ethanol as ethyl esters can partly explain the higher yields in the ethanol based systems. This observation highlights the challenge of estimating the mass balance in the ethanol containing reaction systems, since measurement of the loss of ethanol from the reaction medium is difficult to perform.

There are some notable differences between small- and large scale bio-oils. 2,3-dihydroxypropyl palmitate (**18**) is among the top 5 peaks in about half of the bio-oils in small scale, which is not reflected in large scale. This suggests a more efficient hydrolysis and/or cross-esterification of triglycerides at large scale, which could be an effect of the stirring. Similar observations can be made for 2-pyrrolidine (**1**) and glycerol (**4**), while the opposite is found for 4-methylphenol (**2**) and dodecan-1-ol (**6**), both of which are more abundant at large scale than at small scale. While lower in abundance, similar variations between scales are found for 3-(4-hydroxyphenyl)propanoic acid (**8**), being found in small scale bio-oils at higher concentrations than at large scale, and for pentadecane-1-ol, which is preferably present in the bio-oils from large scale. The aliphatic alcohols can be viewed as products from reduction of fatty acids, which again would support a more efficient reaction at large-scale condition.

**Figure 10:**
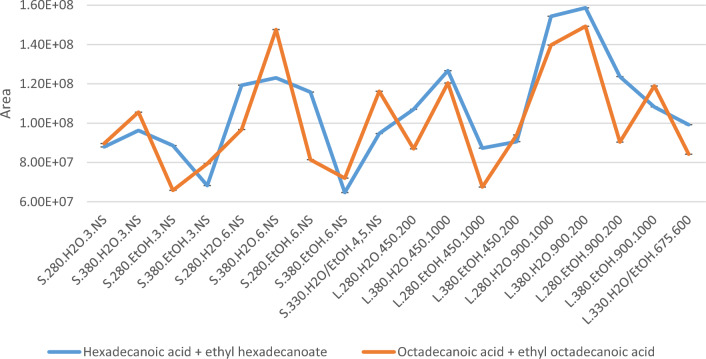
Total peak areas of hexadecanoic acid and ethyl hexadecanoate (blue) and octadecanoic acid and ethyl octadecanoate (orange) from GC–MS.

Based on these results, it could be possible to adjust the reaction factors to increase the desired product compound(s). For instance, if the aim is to achieve the highest possible content of fatty acids for further use, e.g. as biodiesel (fatty acid methyl esters), the concentration of hexadecenoic acid and ethyl hexadecanoate are highest in the bio-oil L.380.H2O.900.200, with L.280.H2O.900.1000 as a close second (shown in Fig. 10, together with similar values of octadecanoic acid and ethyl octadecanoate). As these are the most abundant fatty acids, they can be considered representative of fatty acids in general. The oils with the highest abundances have a lower total oil yield (Fig. 1), but still come out on top regarding the fatty acid contents after adjusting for the yields.

## Conclusion

This study has been performed to better understand the effects of several common variables when upscaling HTL of digested sewage sludge from small scale (25 mL reactors) to bench scale (5.3 L reactor volume). Promising results are found as the experimental design used in this study shows a maximum oil yield within the experimental domain of 67 wt% at large scale (L.280.EtOH.450.1000) and 73 wt% in small scale (S.380.EtOH.6.NS). These values are higher than previously reported in the literature. In addition, a clear trend is seen in the stirred experiments in which decreasing the reaction temperature within the explored range is advantageous to gain higher oil yields if stirring is set to a high rate. This increases the energy efficiency as stirring requires less energy than heating, providing promising results for further upscaling and industrialisation. Compared to the feedstock, a high H/C-ratio is sustained within the bio-oil, while the O/C-ratio is reduced, and the energy recovery relative to the DSS feedstock passes 70% for the high-yield experiments. Regarding the chemical composition of the oil, a total of 18 compounds have been evaluated, with fatty acids and their derivatives providing the largest signals in the GC–MS chromatograms. Previous work has identified a number of compounds within the bio-oil^23^, but in this study only a semi-quantitative analysis is performed for comparison of oils that are made at different reaction conditions. The highest peaks are identified as fatty acids, including all derivatives, and could be separated from the remaining oil in future studies, for further conversion into biodiesel.

The results from this study strongly encourage further work focussing on upscaling and industrial application due to both promising oil yields, the observation of higher oil yields at lower temperatures, and identifying the most abundant compounds within the oil to be fatty acids.

### Supplementary Information

Supplementary Table S1.

## Data Availability

All the data is included in the paper and the supplementary material.
