# Supplementary material for: Exploring hydrothermal liquefaction (HTL) of digested sewage sludge (DSS) at 5.3 L and 0.025 L bench scale using experimental design
Source: Sci Rep. 2023 Nov 1;13:18806. doi: 10.1038/s41598-023-45957-9 (PMC10620431; doi:10.1038/s41598-023-45957-9)
Supplement: Supplementary file 1 — Supplementary Table S1. [file 41598_2023_45957_MOESM1_ESM.docx]

**Supplementary material:**

**Table S1.** Raw data for PLS

|  | Reactor size | Temperature [^o^C] | Medium | Filling rate [mL]^a)^ | Stirring rate [rpm] | Y(oil, *daf*) [wt%] |
| --- | --- | --- | --- | --- | --- | --- |
| S.280.H2O.3.NS | Small | 280 | H_2_O | 3 | 0 | 33.9 |
| S.380.H2O.3.NS | Small | 380 | H_2_O | 3 | 0 | 44.4 |
| S.280.EtOH.3.NS | Small | 280 | EtOH | 3 | 0 | 47.0 |
| S.380.EtOH.3.NS | Small | 380 | EtOH | 3 | 0 | 59.4 |
| S.280.H2O.6.NS | Small | 280 | H_2_O | 6 | 0 | 28.8 |
| S.380.H2O.6.NS | Small | 380 | H_2_O | 6 | 0 | 34.1 |
| S.280.EtOH.6.NS | Small | 280 | EtOH | 6 | 0 | 50.0 |
| S.380.EtOH.6.NS | Small | 380 | EtOH | 6 | 0 | 73.1 |
| S.330.H2O/EtOH.4,5.NS | Small | 330 | H_2_O/EtOH | 4.5 | 0 | 52.1 |
| L.280.H2O.450.200 | Large | 280 | H_2_O | 450 | 200 | 29.1 |
| L.380.H2O.450.1000 | Large | 380 | H_2_O | 450 | 1000 | 37.0 |
| L.280.EtOH.450.1000 | Large | 280 | EtOH | 450 | 1000 | 58.9 |
| L.380.EtOH.450.200 | Large | 380 | EtOH | 450 | 200 | 50.4 |
| L.280.H2O.900.1000 | Large | 280 | H_2_O | 900 | 1000 | 39.3 |
| L.380.H2O.900.200 | Large | 380 | H_2_O | 900 | 200 | 36.4 |
| L.280.EtOH.900.200 | Large | 280 | EtOH | 900 | 200 | 48.2 |
| L.380.EtOH.900.1000 | Large | 380 | EtOH | 900 | 1000 | 54.1 |
| L.330.H2O/EtOH.675.600 | Large | 330 | H_2_O/EtOH | 600 | 600 | 52.8 |

^a)^ The filling rate indicates the amount of the given medium is added. Digested sewage sludge and formic acid is additional, at fixed amounts
